# Structure-based engineering of heparinase I with improved specific activity for degrading heparin

**DOI:** 10.1186/s12896-019-0553-3

**Published:** 2019-08-09

**Authors:** Chuan Zhang, Bao-Cheng Yang, Wen-Ting Liu, Zhong-Yuan Li, Ya-Jian Song, Tong-Cun Zhang, Xue-Gang Luo

**Affiliations:** 1 Key Laboratory of Industrial Fermentation Microbiology of the Ministry of Education & Tianjin Key Lab of Industrial Microbiology, College of Biotechnology, Tianjin University of Science and Technology, Tianjin, 300457 China; 2 State Key Laboratory of Food Nutrition and Safety, Tianjin, 300457 China

**Keywords:** Heparinase I, Specific activity, Site-directed mutagenesis, Homology modeling, Molecular docking

## Abstract

**Background:**

Heparinase I from *Pedobacter heparinus* (Ph-HepI), which specifically cleaves heparin and heparan sulfate, is one of the most extensively studied glycosaminoglycan lyases. Enzymatic degradation of heparin by heparin lyases not only largely facilitates heparin structural analysis but also showed great potential to produce low-molecular-weight heparin (LMWH) in an environmentally friendly way. However, industrial applications of Ph-HepI have been limited by their poor yield and enzyme activity. In this work, we improve the specific enzyme activity of Ph-HepI based on homology modeling, multiple sequence alignment, molecular docking and site-directed mutagenesis.

**Results:**

Three mutations (S169D, A259D, S169D/A259D) exhibited a 50.18, 40.43, and 122.05% increase in the specific enzyme activity and a 91.67, 108.33, and 75% increase in the yield, respectively. The catalytic efficiencies (*k*_*cat*_/*K*_*m*_) of the mutanted enzymes S169D, A259D, and S169D/A259D were higher than those of the wild-type enzyme by 275, 164, and 406%, respectively. Mass spectrometry and activity detection showed the enzyme degradation products were in line with the standards of the European Pharmacopoeia. Protein structure analysis showed that hydrogen bonds and ionic bonds were important factors for improving specific enzyme activity and yield.

**Conclusions:**

We found that the mutant S169D/A259D had more industrial application value than the wild-type enzyme due to molecular modifications. Our results provide a new strategy to increase the catalytic efficiency of other heparinases.

**Electronic supplementary material:**

The online version of this article (10.1186/s12896-019-0553-3) contains supplementary material, which is available to authorized users.

## Background

Heparin is a mixed sulfated polysaccharide with specific properties and dispersions. It was originally found in the liver, so it was named heparin. Heparin levels are relatively high in the lungs, ileum and skin of most species analyzed [1]. Heparinase acts directly upon heparin, yielding 52% of a trisulfated disaccharide (O-(α-L-ido-4-enepyranosyluronic acid 2-sulfate)-(1–4)-2-sulfoamino-2-deoxy-D-glucose 6-sulfate) and 40% a tetrasaccharide, in addition to small amounts of hexa- and disaccharides [2]. Although heparin is widely used in medicine, its actual biological role remains unknown [3]. The polysaccharide compounds have good activity on immune regulation, anti-tumor, anti-coagulation, and anti-inflammatory properties and have been applied to the pharmaceutical industry [4, 5]. Low-molecular-heparin (LMWH) is a small fraction of heparin produced by physical, chemical or enzymatic hydrolysis of heparin. Compared with normal-molecular-weight heparin, low-molecular-weight heparin has less anti-factor IIa activity and a lower risk of bleeding [6]. Currently, there are physical, chemical, biological, and synthetic methods for preparing low-molecular-weight heparin. Among these methods, the bioenzymatic method has become an emerging method due to its advantages of mild conditions, strong selectivity and low pollution.

In general, heparinase is an enzyme that degrade heparin by lysis. At present, most research focuses on microorganism-derived heparinases [7]. There are three variants of heparinase (HepI, −II, −III), which differ in the catalytic site and enzymatic activity. Heparinase I (HepI), first isolated from *Pedobacter heparinus*. is the polysaccharide lyase that has been most extensively used to study the physiological roles of heparin-like complex polysaccharides. Heparinase I (HepI) is a polysaccharide lyase that depolymerizes heparin and heparan sulfate to produce unsaturated disaccharides and oligosaccharides by the β-elimination mechanism [8, 9].

Moreover, heparinase can be widely used to produce low-molecular-heparins [10, 11]. LMWH has more stable and predictable anticoagulation activity than unfractionated heparin, and is widely used in the prevention and treatment of venous thromboembolism and in the treatment of myocardial infarction [8]. In addition, HepI is the most important tool for analyzing heparin structure [12] and eliminating heparin in human blood [13, 14]. However, whether in laboratory-scale heparin analysis or in industrial-scale low-molecular-weight heparin production, the original manufacture of *P. heparinus* HepI is costly, which largely hinders the application of HepI [15]. Although HepI has been heterologously expressed in *Escherichia coli*, the yield was very low due to the presence of inclusion bodies [16]. The high price, low yield and low activity of HepI greatly hinder its industrial application.

Using homology modeling, multiple sequence alignment, and site-directed mutagenesis, many enzymes have been modified to improve the activity, specificity and thermostability [17–20]. However, there are still no reports on the structure-based molecular evolution of HepI. Therefore, we proposed a rational design method based on the structure of Ph-HepI from *P. heparinus* to improve its catalytic activity. Through docking a substrate and a calcium ion into the Ph-HepI model structure, analyzing related residues and multiple sequence alignment with Bt-HepI, two residues that might increase catalytic activity were selected. Mutation analysis of key amino acid residues (Ala259 and Ser169) in the loops (251–280 and 165–172) that constitute the substrate cover site and calcium binding site of the Ph-HepI was performed. To the best of our knowledge, this is the first report on improving the specific enzyme activity of HepI by protein structural engineering.

## Methods

### Materials

The cloning strain and the expression strain were *E. coli* DH5α and *E. coli* Rosetta (DE3), respectively. Plasmid pE-SUMO was employed as an expression vector with a 6 × His tag and a SUMO tag upstream of the multiple cloning sites. Heparin sodium salt (185 U/mg) was purchased from Solarbio company (Beijing, China). DNA polymerases, restriction enzymes, and T4 ligase were purchased from ThermoFisher (USA). The His GraviTrap TALON was purchased from GE Health (Uppsala,Sweden).

### Construction of HepI expression plasmid

The coding gene sequence of *P. heparinus* HepI (GenBank accession no. AAA24920.1) was optimized according to the codon usage table of *E. coli* (GenBank accession no. MK229006) and was synthesized by Genewiz (Beijing). The hepI gene (1089 bp) flanked by *Bsa* I and *Bam* HI sites was amplified by PCR using the forward primer 5′-GCGGTCTCGGTGGCGGTGGCAGTGATGATGATAAACAGCA-3′ and a reverse primer 5′-TCGGATCCTTAACGAGCAGTTTCGGAGTAAC-3′. A flexible peptide linker (G4S) sequence and a bovine enterokinase site (D4K) sequence were also inserted at the C-terminus of Ph-HepI. The PCR fragment was inserted into the *Bsa* I and *Bam* HI sites of the pE-SUMO vector.

### Sequence alignment and homology modeling of HepI

An initial sequence alignment with *P. heparinus* HepI and *Bacteroides thetaiotaomicron* HepI (GenBank accession no. AAO79780.1) (Bt-HepI) was performed by ClustalW [21], and the resulting figure was prepared with ESPript 3.0 [22]. A phylogenetic tree of Ph-HepI and other characterized polysaccharide lyases from the PL13 family (http://www.cazy.org) was constructed by MEGA 6.0 using the neighbor-joining method, and the boot-strap value was set as 1000. A 3D model structure of *P. heparinus* HepI was built using SWISS-MODEL (https://www.swissmodel.expasy.org/) based on the known crystal structure of *B. thetaiotaomicron* HepI (Bt-HepI, PDB code: 3IKW) [23].

### Site-directed mutagenesis

The recombinant plasmid was PCR-amplified with mutagenic oligonucleotides using a Mut Express II Fast Mutagenesis Kit (Vazyme, Nanjing). The mutant primer sequences are shown in Table 1. PCRs were performed using *Phanta Max Super-Fidelity DNA polymerase* for 33 cycles. Each 50 μL reaction mixture consisted of 2 μL of plasmid carrying the HepI, 25 μL of 2× Max buffer, 1 μL of dNTP Mix (10 mM each), 2 μL of 10 μM forward primer, 2 μL of 10 μM reverse primer, 1 μL of *Phanta Max Super-Fidelity DNA polymerase,* and ddH_2_O up to 50 μL. Thermal cycling conditions were 95 °C for 15 s followed by 30 s of 95 °C for 15 s, 62 °C for 15 s, and 72 °C for 2 min, and a final step at 72 °C for 5 min. The PCR product was digested using *Dpn* I for 2 h at 37 °C. The reaction mixture consisted of 50 μL of amplification products and 1 μL of *Dpn* I*.* All gel-purified reaction products were recycled using the Gel Extraction Kit. The *Dpn* I digestion product was ligated into circular plasmids using Exnase II for 30 min at 37 °C. The reaction mixture consisted of 200 ng *Dpn* I digestion product, 4 μL of 5× CE II Buffer, 2 μL of Exnase II, and ddH_2_O up to 20 μL. These plasmids were sequenced correctly (GENEWIZ, Suzhou) and transformed into expressed strains (*E. coli* Rosetta (DE3)).

**Table 1:** Oligonucleotide primer pairs used for the construction of mutant enzymes

S. no.	Amino acid change	Primer sequences^a^
1	S169D	5′-CAGTGGCACGGTGCTCCGGAT(GCT)CGTACTCTTGTTGCTACT − 3′ 5′-AGTAGCAACAAGAGTACGATC(AGC)CGGAGCACCGTGCCACTG − 3′
2	A259D	5′-GTGGCTTACTGATAAAGAT(GAA)GATCGTAACAACGCTA-3′ 5′-TAGCGTTGTTACGATCATC(TTC)TTTATCAGTAAGCCAC-3′

^a^Base changes in the relevant triplets are shown in bold and underlined. The original triplets are given in the parentheses

### Enzyme expression and purification

The recombinant strain carried plasmid pE-SUMO-HepI and was cultured for 12–15 h at 37 °C on LB agar plates containing 34 μg/mL chloramphenicol and 50 μg/mL kanamycin. The seed culture (1%) was then inoculated into 100 mL of LB medium with antibiotics and cultivated at 37 °C and 220 rpm until the optical density at 600 nm (OD_600_) reached 0.6. The cells were induced with 0.6 mM isopropyl-β-D-thiogalactopyranoside (IPTG) at 30 °C for 9 h. Cells were harvested by centrifugation at 8000 rpm for 10 min at 4 °C. Cells were washed twice with Tris–HCl buffer (20 mM Tris, 200 mM NaCl, 5 mM CaCl_2_, pH 7.4) and resuspended in 30 mL of the same buffer, followed by sonication (sonication 3 s, pause 4 s, 300 W) on ice for 10 min to disrupt the cells. Cell debris was separated by centrifugation at 12,000 rpm for 20 min at 4 °C. The expressed HepI was the soluble protein in the supernatant [24] and analyzed by resolving 12% SDS-PAGE. The target proteins stained with Coomassie Brilliant Blue R-250 were visualized by an Odyssey Infrared Imaging System (Gene Company, Beijing, China). The supernatant containing the target protein was filtered using a 0.45 mm filter membrane and then loaded onto a Hi-Trap metal-chelating affinity column. The column was washed with 10 mL of buffer (50 mM Tris, 300 mM NaCl, 5 mM CaCl_2_, 50 mM imidazole, pH 7.4) to wash away protein. Finally, 3–5 mL of elution buffer (50 mM Tris, 300 mM NaCl, 10 mMCaCl_2_, 150 mM imidazole, pH 7.4) was applied to obtain the purified SUMO-HepI. The expression yield of HepI was analyzed by SDS-PAGE, and measured by Bradford’s method [25].

### Determination of enzyme activity and kinetic parameters

The heparinase I activity was measured by the 232 nm method [26]. The reaction was implemented at 30 °C using heparin as the substrate in reaction buffer (containing 25 g/L heparin, 50 mM CH_3_COONa, 5 mM Ca(CH_3_COO)_2_ pH 7.4). Heparin degradation was detected by the UV absorbance at 232 nm on a UV-3200 spectrophotometer (Mapada, Shanghai) and the activity was calculated using a molar extinction coefficient of 3800 M^− 1^ cm^− 1^. One international unit (IU) was defined as the amount of protein that could form 1 μmol unsaturated uronic acid per minute at 30 °C.

The kinetic parameters (*K*_*m*_, *Vmax*, *k*_*cat*_ and *k*_*cat*_/*K*_*m*_) of wild-type and mutated Ph-HepI were determined at 37 °C and pH 7.4. The *K*m and *V*max were calculated with Eadie-Hofstee plots.

### Effects of temperature, pH and concentration of Ca^2+^ on enzyme activity

The optimal temperature of the pure enzyme was measured by assaying the enzyme activity at various temperatures (25 °C, 29 °C, 33 °C, 37 °C, 41 °C, 45 °C). Wild-type enzyme activity at 25 °C was defined as 100%, and the relative enzyme activity of the mutants was calculated based on wild-type enzyme activity. The optimal pH of the pure enzyme was measured by assaying the enzyme activity at various pH values within the range of 4–10. Wild-type enzyme activity under pH 4 was defined as 100%, and relative enzyme activity of the mutants was calculated based on wild-type enzyme activity. The optimal concentrations of Ca^2+^ for the pure enzyme was measured by assaying the enzyme activity at various concentrations of Ca^2+^ (0 mM, 5 mM, 10 mM, 50 mM, 100 mM, 200 mM). Wild-type enzyme activity under concentrations of 0 mM Ca^2+^ was defined as 100%, and relative enzyme activity of the mutants was calculated based on wild-type enzyme activity.

### Mass spectrometry analysis of the product

Analysis of degraded low-molecular-weight heparin used oligosaccharide time-of-flight mass spectrometry (MALDI-TOF-MS) [27]. First, a low-molecular-weight heparin was irradiated with a laser to form a film cocrystallized with the matrix, and then the matrix was absorbed into the energy by the laser and then transmitted to the biomolecule. The main principle of time-of-flight mass spectrometry is that the ions are accelerated through the flight path by an electric field, and the mass-to-charge ratio (M/Z) of the ions ionized by the sample is determined from their time of arrival at the detector. Target plate: ground steel target. Matrix solution: 2,5-DHB (20 mg/L, T30 dissolved). Matrix additive: 1 mM NaCl. The sample was dissolved using 30% acetonitrile (1 mg/mL). Sample Preparation: The solution included 1 μL of sample solution and 1 μL of matrix solution and was naturally dried on the sample target.

### Detection of anti-Xa and anti-IIa activity of the product

LMWH anti-Xa and anti-IIa factor activity detection referred to the European Pharmacopoeia [28]. This method compares the anti-thrombin activity of a sample with the LMWH standard in vitro to determine the ability of the test sample to accelerate the inhibition of factor Xa and factor IIa. The reagents used were purchased from Shanghai Boatman Biotech.

REAGENT 1: Human antithrombin (AT): Each vial was reconstituted with exactly 1 mL of distilled water. It was shaken thoroughly until complete dissolution of the contents (vortex). The contents homogenized for 30 min at room temperature (18–25 °C) while shaking the vial from time to time. Just before use, the sample was diluted 1:5 with R4 buffer. REAGENT 2: Factor Xa/factor IIa: Each vial was reconstituted with exactly 1 mL of distilled water and shaken thoroughly until complete dissolution of the contents (vortex). The contents homogenized for 30 min at room temperature (18–25 °C) while shaking the vial from time to time. Just before use, each sample was diluted 1:5 with R4 buffer. REAGENT 3: Factor Xa/factor IIa specific chromogenic substrate: Each vial was reconstituted with exactly 5 mL of distilled water and shaken thoroughly until complete dissolution of the contents (vortex). The contents homogenized for 30 min at room temperature (18–25 °C) while shaking the vial from time to time (vortex). REAGENT 4: Assay Reaction Buffer at pH 8.40: Ready-to-use vial of 25 ml. It was shaken before use.

The reaction was carried out in a microplate. Manual method: We added 40 μL of heparinized sample (at 1:10 dilution) and 40 μL of R1, mixed, and incubated at 37 °C for 2 min. We then introduced 40 μL of R2, mixed, and incubated at 37 °C for exactly 2 min (stage 1). Next, we introduced 40 μL of R3, mixed, and incubated at 37 °C for exactly 2 min (stage 2). The reaction was stopped by introducing 80 μL of citric acid (20 g/L). We mixed and measured the absorbance at 405 nm against the corresponding blank.

### Statistical analysis

All experiments were performed independently at least three times, and the results are expressed as the mean ± standard deviation. The T-test was used for statistical analysis.

## Results

### Sequence analysis and homology modeling

**Fig. 1:**
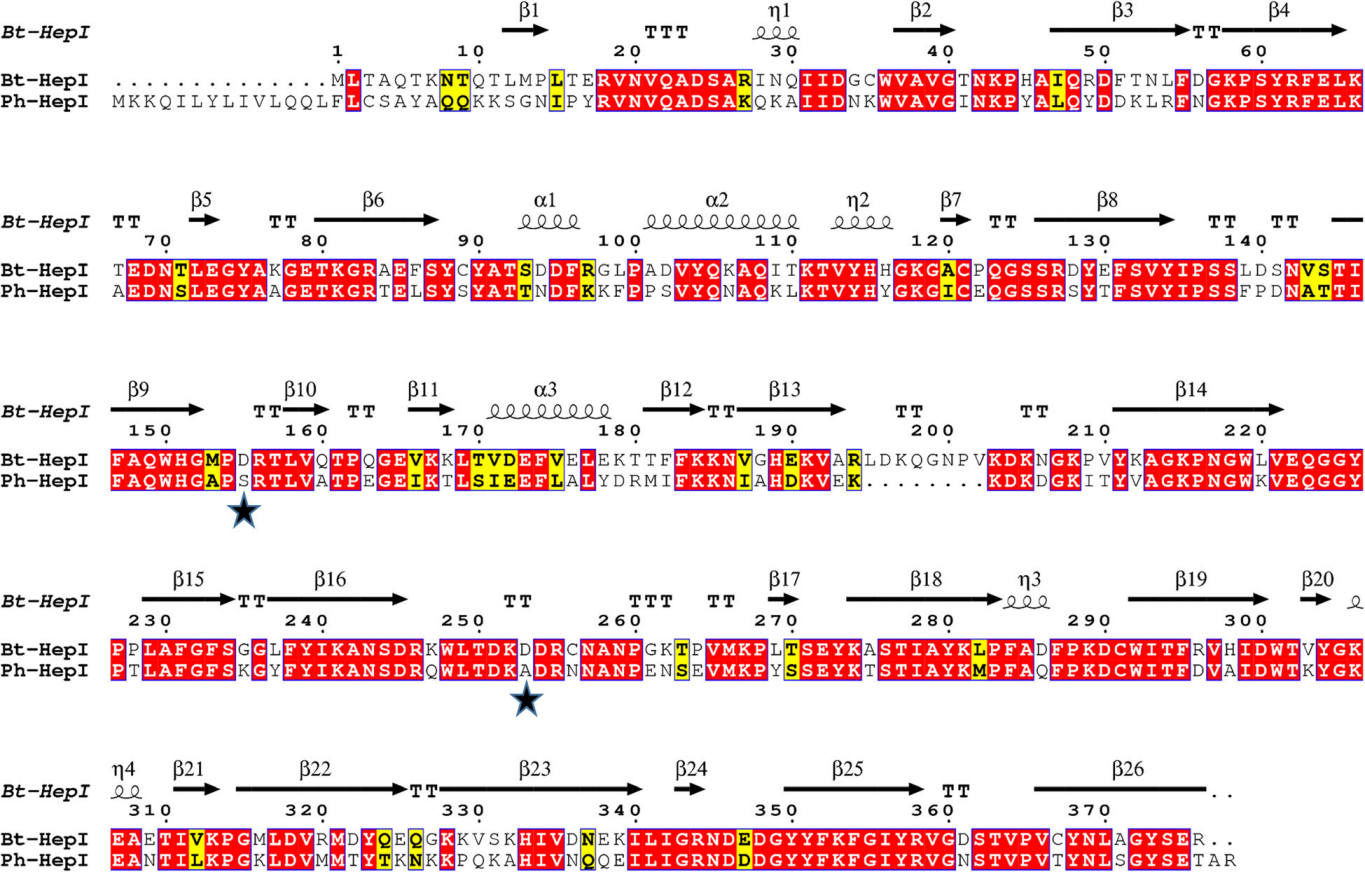
Structural sequence alignment of Bt-HepI and Ph-HepI. Residues forming the secondary structures are highlighted in the Bt-HepI sequence. Identical and similar amino acid residues are shown by white letters on a red background and black letters on a yellow background, respectively. Two different amino acids that are not conserved in Ph-HepI and Bt-HepI are indicated by black stars

**Fig. 2:**
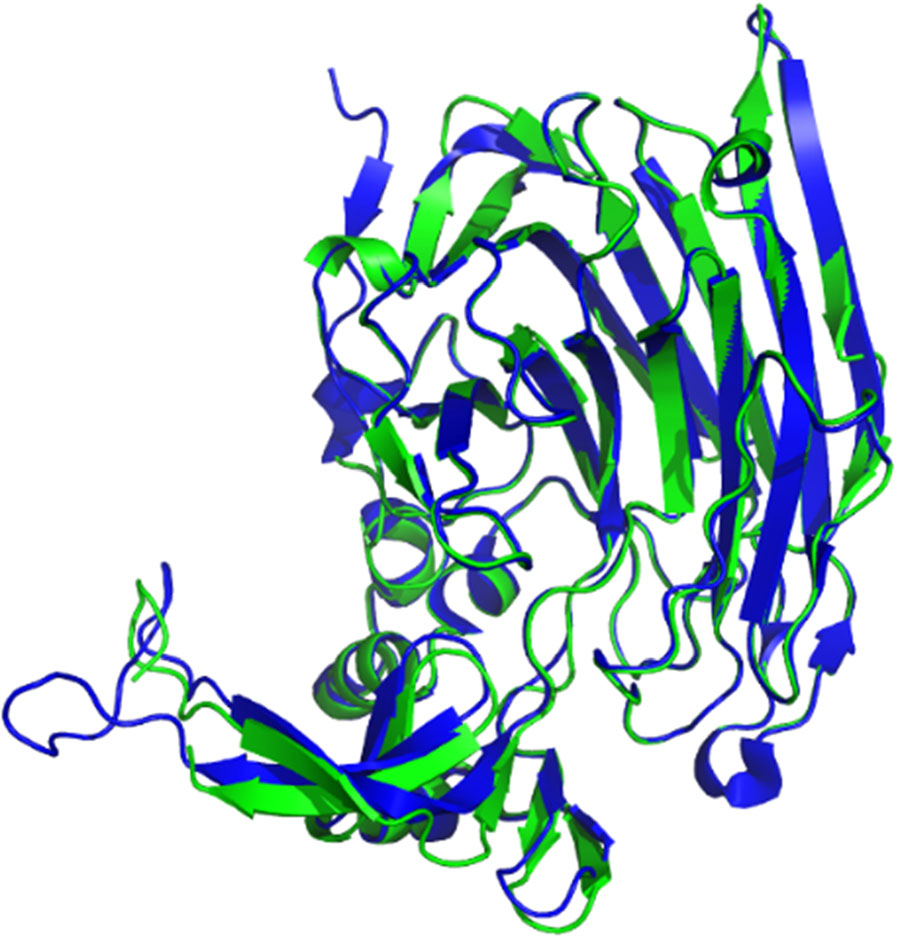
The overall structure of Ph-HepI. Structure comparison of Ph-HepI (green) is superimposed with the template from *Bacteroides thetaiotaomicron* (blue; PDB ID: 3ikw)

Protein sequence analysis showed that Ph-HepI shared 62.5% identity with the *B. thetaiotaomicron* HepI and multiple sequence alignment (MSA) of the two heparinase I enzymes is shown in (Fig. 1). To explore the key sites of Ph-HepI, a homology model of the protein was built using SWISS-MODEL (Fig. 2) according to the known crystal structure of the *B. thetaiotaomicron* HepI. Additional file 1: Figure S1 shows the phylogenetic tree of Ph-HepI and the characterized polysaccharide lyases from the PL13 family. It can be seen that most characterized PL13 family polysaccharide lyases are from bacteria. Additional file 2: Figure S2 shows that the 3D model structure of *P. heparinus* HepI was reasonable. Residues in most favored regions were over 90% conserved. The stereochemistry of the structure was analyzed using structural analysis and verification server of the University of California, Los Angeles (http://services.mbi.ucla.edu/SAVES/). Finally, all structural figures were generated with PyMOL (www.pymol.org) [29].

### Enzymatic interactions related residues and selection of mutational sites

**Fig. 3:**
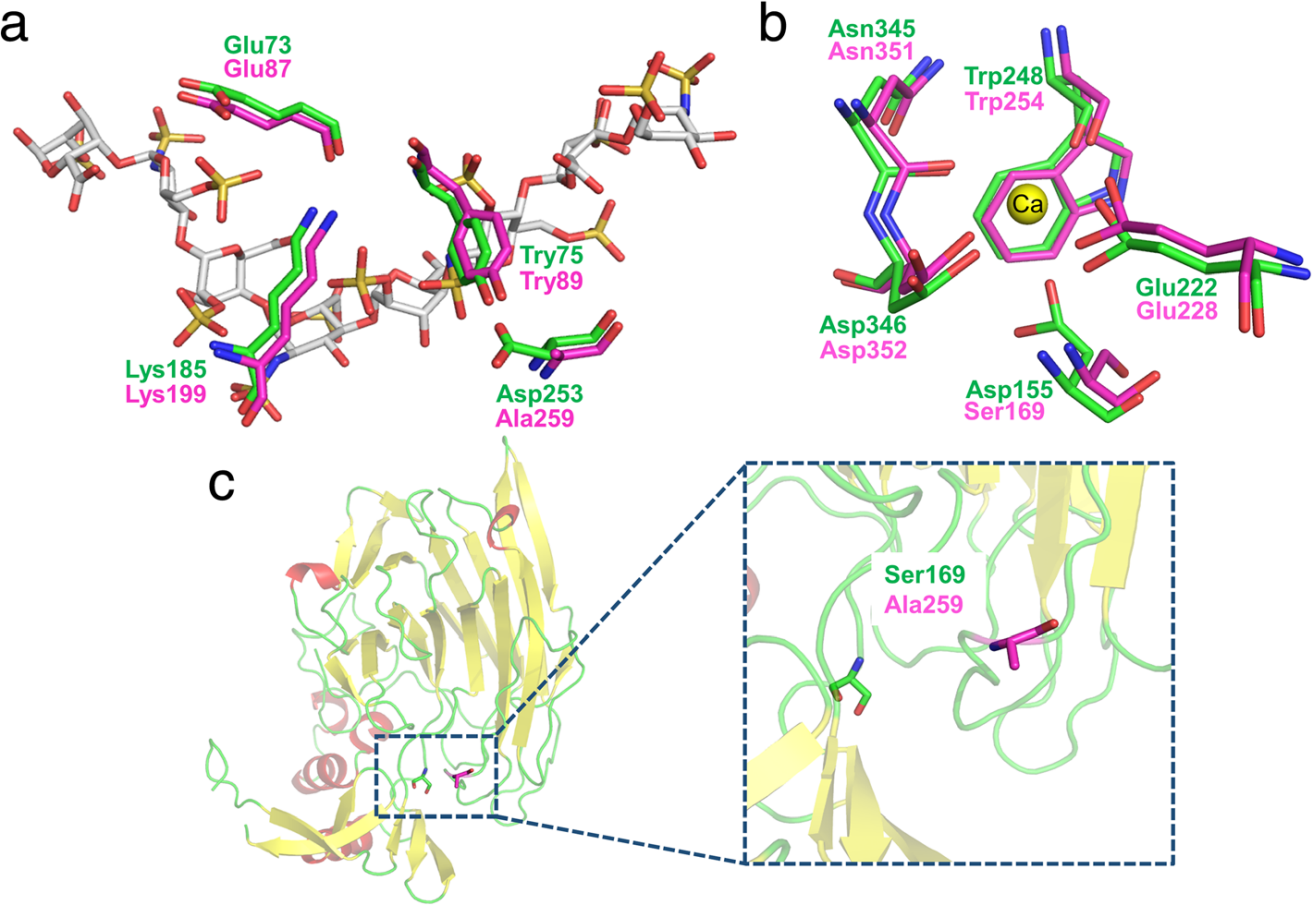
Substrate recognition and calcium ion binding interaction sites. (**a**) The substrate is shown as sticks in gray. Residues from template and Ph-HepI are shown in green and magenta, respectively. (**b**) The calcium ion is shown as a yellow sphere. Residues from template and Ph-HepI are shown in green and magenta, respectively. (**c**) Cartoon representation of the structure model of Ph-HepI. The two residues (Ser169 and Ala259) in the Ph-HepI are indicated by markers in green and magenta, respectively. Two amino acids in Ph-HepI are different from template (Ser169, Ala259)

It has been reported that residues D155, E222, W248, N345, and D346 in Bt-HepI are involved in calcium ion binding [23]. Based on the alignment assay, these residues in Bt-HepI were corresponded to S169, E228, W254, N351, and D352 in Ph-HepI (Fig. 3a). In the template, several amino acid residues formed a perfect lid that covered the substrate, including E73, Y75, K185, and D253, which correspond to E87, Y89, K199, and A259 in Ph-HepI (Fig. 3b). The amino acid residues 169 and 259 in Ph-HepI were different from the corresponding residues in Bt-HepI (Fig. 3c). Other residues in Ph-HepI, which also interacted with the substrate, shared no obvious counterparts in the template. The presence of a Ca^2+^ ion is crucial for heparinase I activity [30, 31]. From molecular docking, a Ca^2+^ binding site was also found in the active pocket of Ph-HepI. In general, the Ca^2+^ ion plays an important role in facilitating bridging between the enzyme and substrate in the complex.

**Fig. 4:**
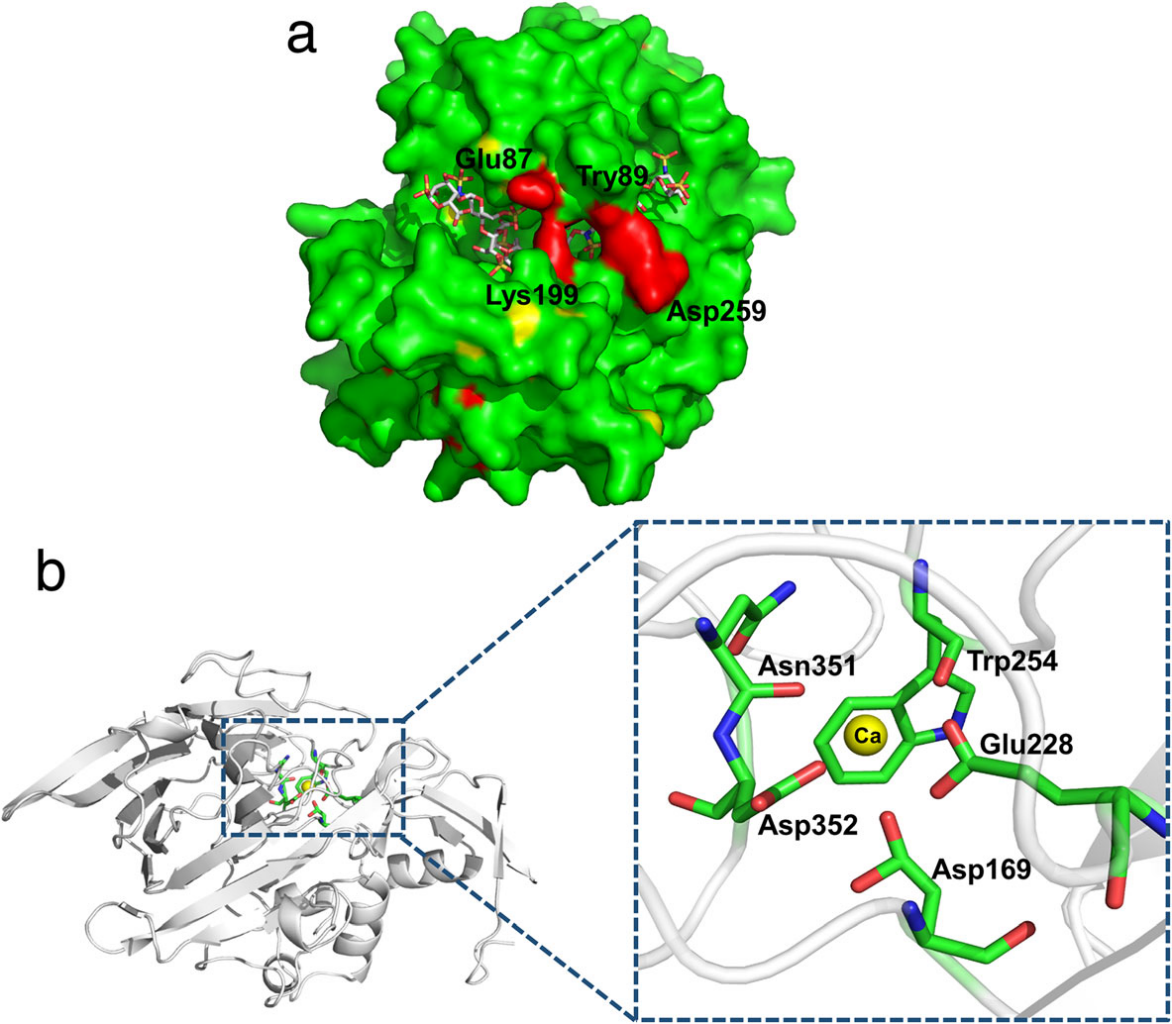
Structures of Ph-HepI bound with heparin and calcium ion. (**a**) The binding of heparin in the positively charged canyon of Ph-HepI-A259D is shown as a surface charge presentation. The similar cover, including four amino acids (Glu87, Tyr89, Lys199, Asp259) on the surface, is shown in red. (**b**) The interaction between Ph-HepI and calcium ion (Ca^2+^). The Ca^2+^ is shown as a yellow sphere. Amino acid residues Asp169, Glu228, Trp254, Asn351, and Asp352, which are essential for the interaction, are shown in sticks

The model structure of Ph-HepI revealed an unusually deep and narrow shape of the substrate tunnel. We performed docking of the structures of Ph-HepI and heparin to further investigate substrate binding (Fig. 4a).. The results showed that there was a perfect lid to cover the substrate, consisting of four amino acid residues (E87, Y89, K199, and A259). Compared with the two enzymes, the residue A259 is different in Ph-HepI. Bipyramidal coordination of Ca^2+^ ion in the Ph-HepI structure among the residues (S169, E228, W254, N351, and D352) involved in the substrate-binding pocket was also discovered (Fig. 4b). Ph-HepI had a similar structure to Bt-hepI, but the amino acid residue S169 was different from the D155 in Bt-HepI. Therefore, to improve the specific enzyme activity of Ph-HepI, three mutants, S169D, A259D, and S169D/A259D, were designed.

### Expression and purification of Ph-HepI mutants

**Fig. 5:**
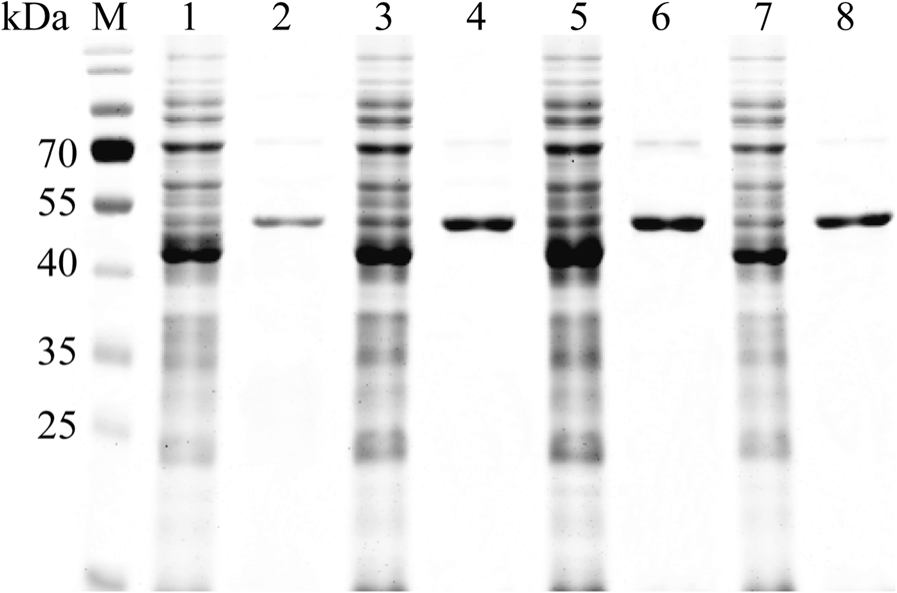
The purified protein was analyzed by 12% SDS-PAGE and stained with Coomassie Brilliant Blue R-250. Lane M—protein marker; lanes 1, 3, 5, 7—wild-type Ph-HepI and S169D, A259D and S169D/A259D mutants are produced by inducing the recombinant bacteria; lanes 2, 4, 6, 8—purified wild-type Ph-HepI and S169D, A259D and S169D/A259D mutants. The band of Ph-HepI protein (53 kDa) is indicated by an arrow

The wild-type and mutant recombinant expression strains were successfully constructed. By analyzing the expression of wild-type and mutant Ph-HepI, it was found that there were different expression levels. Three mutants of Ph-HepI showed soluble expression, which was used to determine enzymatic properties. The purification of Ph-HepI wild-type and mutants was analyzed by 12% SDS-PAGE (Fig. 5). Obviously, the amount of protein expressed was significantly increased after the mutation. The protein concentrations of Ph-HepI wild-type, S169D, A259D and S169D/A259D were 0.12, 0.23, 0.25, and 0.21 mg/mL by quantitative protein and gray-level analysis, respectively.

### Enzymatic characterization and kinetic parameters of Ph-HepI mutants

**Fig. 6:**
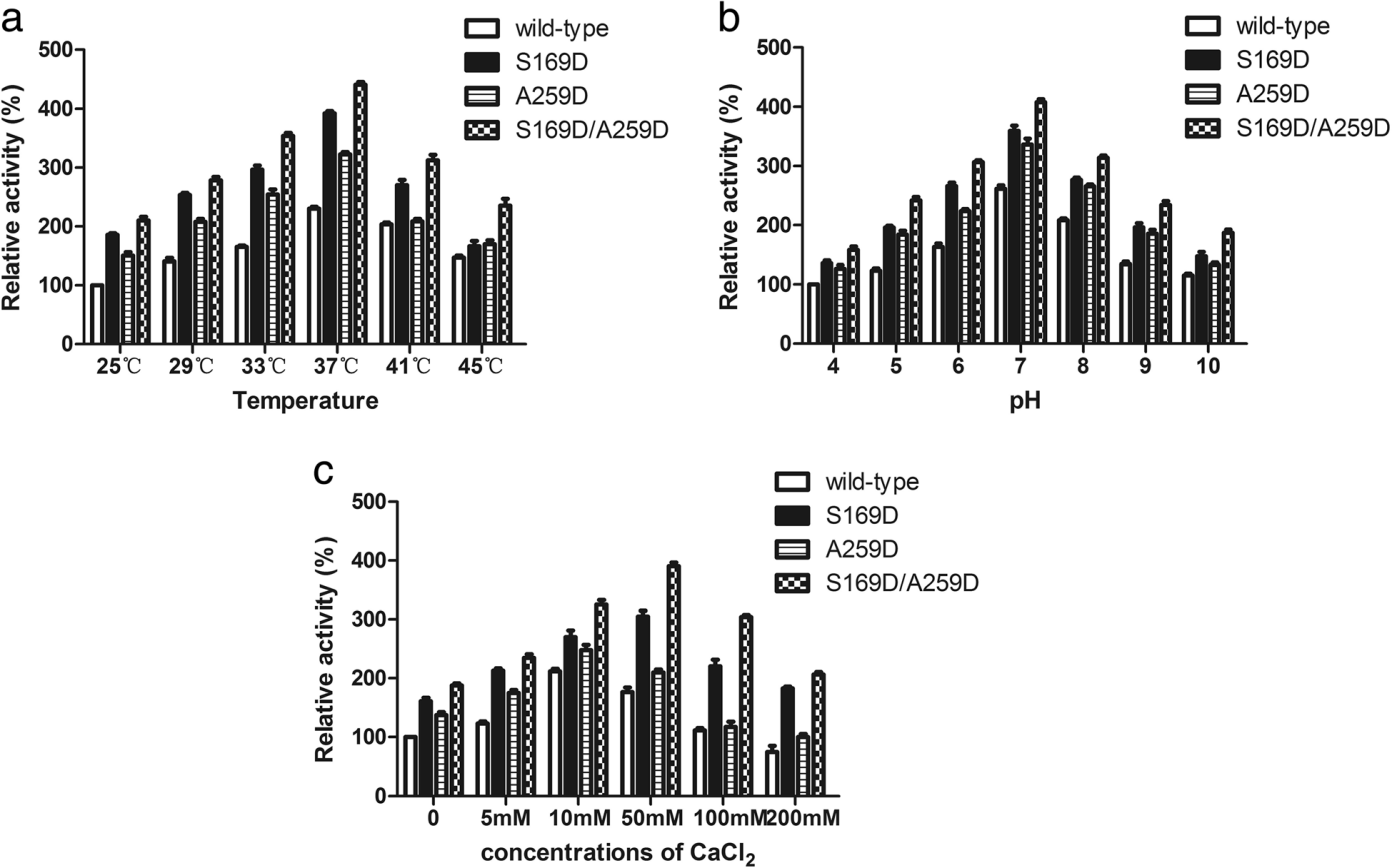
Effects of temperature (**a**), pH (**b**) and concentration of Ca^2+^ (**c**) on the activities of mutated Ph-HepI

To investigate the effects of on the bioactivity of the mutation, enzymatic characteristics of wild-type and mutated enzymes were measured. As shown in Fig. 6, amino acid mutations did not alter the optimal reaction temperature or pH of enzymes, and they all had an optimal temperature and pH of 37 °C and 7, respectively (Fig. 6a-b). However, the optimal concentration of Ca^2+^ was changed, and the optimal concentration of Ca^2+^ for wild-type and A259D mutations was 10 mM, but the optimal concentration of S169D and S169D/A259D mutations was 50 mM (Fig. 6c). The reason for this phenomenon might be that the amino acid at position 169 is in the binding region of Ca^2+^, the Asp has a longer side chain than Ser, and it is an acidic amino acid with a stronger electron cloud density. When the Ser was mutated to Asp, the binding of the enzyme to Ca^2+^ was enhanced.

As shown in Table 2, compared with the wild-type enzyme, the *K*_*m*_ values of the mutant enzymes S169D, A259D, and S169D/A259D were lower than the wild-type enzyme by 68, 58 and 73%, respectively. Furthermore, compared with the wild-type enzyme, the catalytic constants (*k*_*cat*_) of the mutated enzymes S169D, A259D, and S169D/A259D were higher than those of the wild-type enzyme by 20, 12, and 31%, respectively. Consequently, the catalytic efficiencies (*k*_*cat*_/*K*_*m*_) of the mutated enzymes S169D, A259D, and S169D/A259D were higher than those of the wild-type enzyme by 275, 164, and 406%, respectively.

**Table 2:** Kinetic parameters of wild-type, mutated and combined mutations Ph-HepI

Enzyme	*K*_*m*_ (mM)	*k*_*cat*_ (s^− 1^)	*k*_*cat*_/K_m_ (s^− 1^ M^− 1^ × 10^− 3^)
Wild type	0.78 ± 0.05	19.31 ± 0.17	24.76
S169D	0.25 ± 0.07	23.19 ± 0.09	92.76
A259D	0.33 ± 0.09	21.56 ± 0.15	65.33
S169D/A259D	0.21 ± 0.06	26.32 ± 0.03	125.33

### Enzymatic activity and structural analysis of mutants

**Fig. 7:**
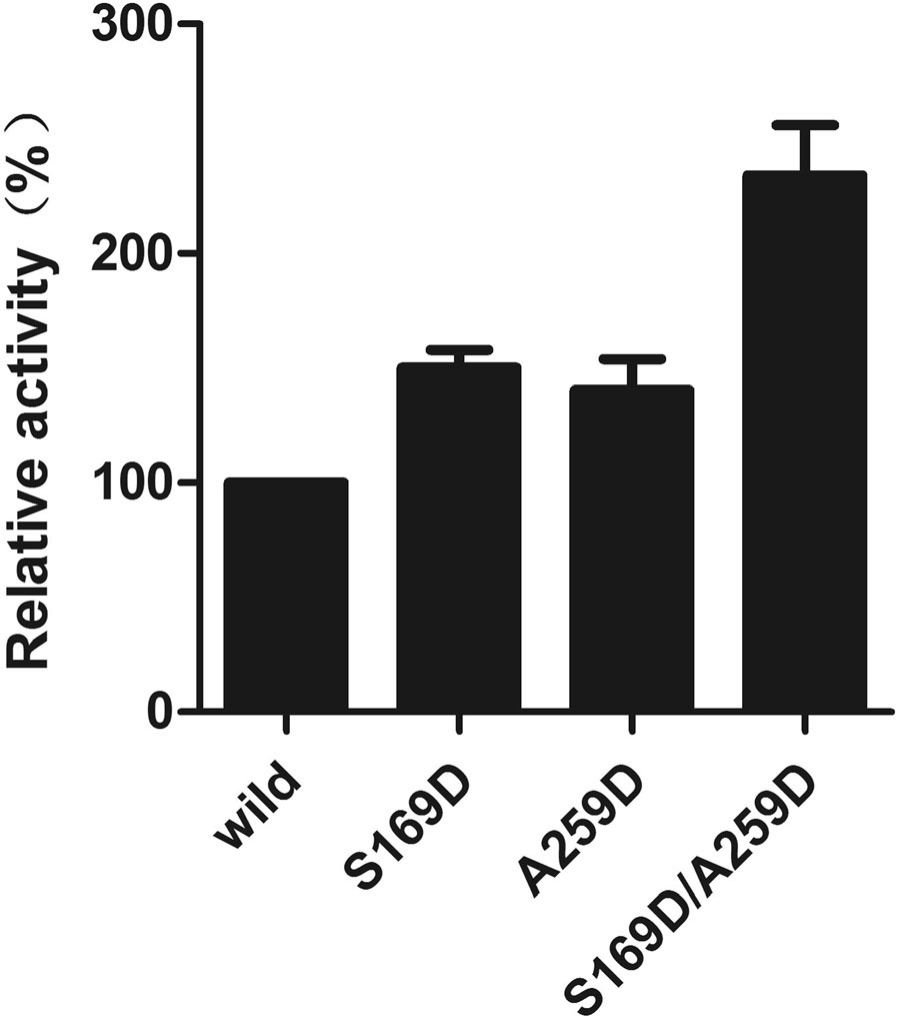
The relative activities of Ph-HepI mutants. The activity of the Ph-HepI WT was taken as 100%. The mutants of Ph-HepI with considerable enzymatic activity are represented as bars. The error bars indicate the standard deviation obtained from three independent experiments

When the purified proteins were obtained, the enzyme activity of the wild-type and three mutants of Ph-HepI proteins were examined. As shown in Fig. 7, the S169D mutation exhibited an obvious improvement of hydrolysis activity on heparin, demonstrating that S169 is crucial for Ph-HepI activity. Another active-site mutation A259D, resulted in obvious improvement of enzymatic activity, showing that this mutation is essential for catalysis. The combined mutation S169D/A259D showed the highest enzyme activity toward heparin. Compared with the wild-type enzyme activity, the specific enzyme activity of mutations S169D, A259D and S169D/A259D increased by 50.18, 40.43, and 122.05% (Fig. 7).

**Fig. 8:**
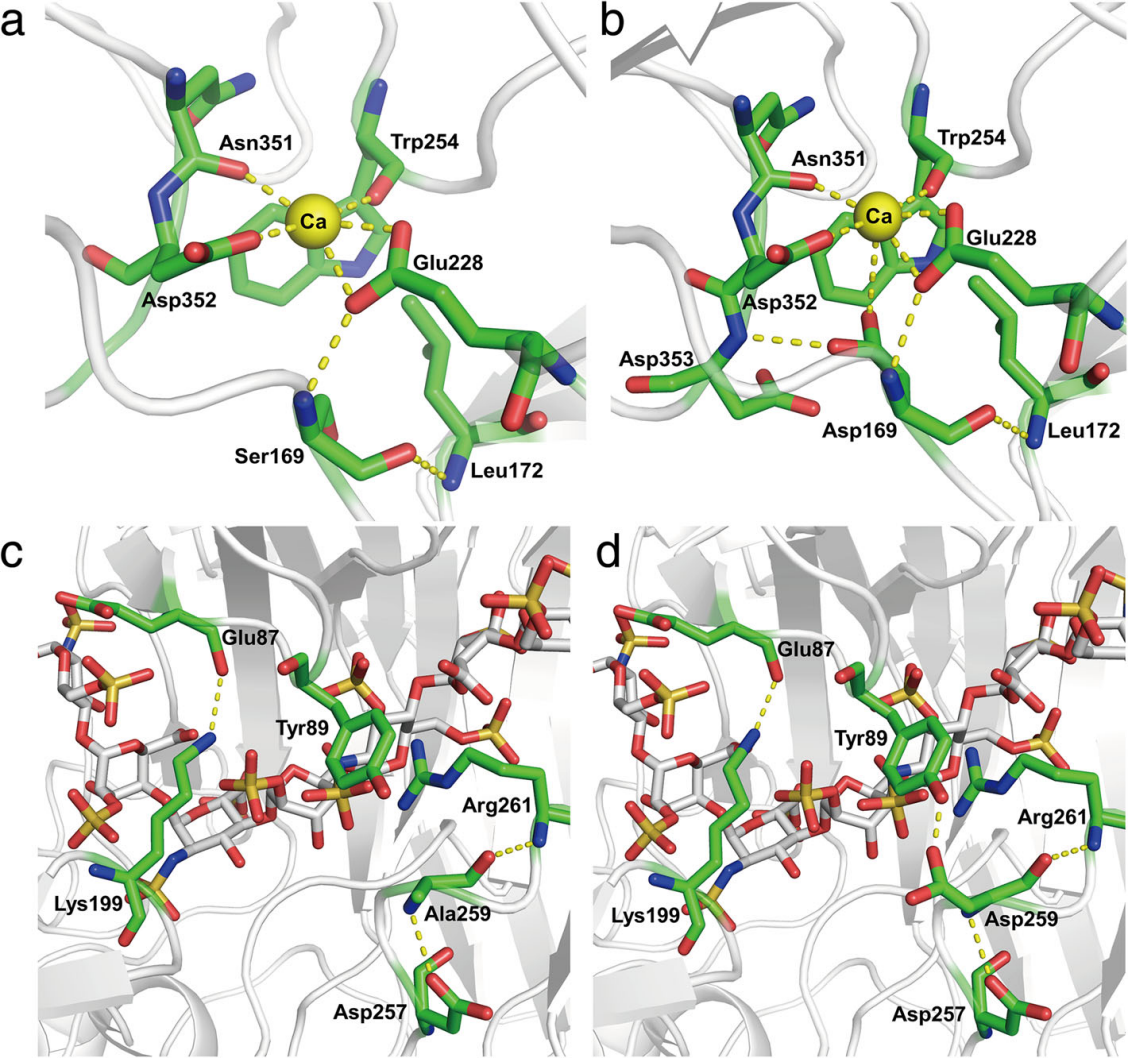
Structural analysis of Ph-HepI wild-type and mutant enzymes. (**a**) In the wild-type, Ser169 forms two hydrogen bonds with Leu172 and Glu228. (**b**) In the mutant S169D, Asp169 forms three hydrogen bonds with adjacent amino acids (Leu172, Glu228, and Asp353) and forms an ionic bond with the calcium ion. (**c**) In the wild-type, Ala259 forms two hydrogen bonds with Asp257 and Arg261. (d) In the mutant A259D, Asp259 forms three hydrogen bonds with Tyr89, Asp257, and Arg261

To understand how mutations affect the catalytic efficiency of enzymes, we analyzed the structure of a series of mutants (S169D, A259D, and S169D/A259D). Asp is an acidic amino acid with a strong negative charge, and its side chain is longer in space than Ser. Therefore, when the amino acid at position 169 was mutated from Ser to Asp, and an ionic bond formed with the calcium ion. The amino acid Asp not only forms an ionic bond with a calcium ion, but also forms a hydrogen bond with the adjacent Asp353. Finally, the Ph-HepI and substrate were more tightly bound, increasing the enzyme’s specific enzyme activity (Fig. 8a-b). Additionally, when the amino acid at position 259 was mutated from Ala to Asp, a hydrogen bond formed between Asp 259 and Try 87. Furthermore, there were four amino acids (Glu87, Lys199, Tyr89, Asp259) above the substrate that formed a similar lid to cover the substrate by forming a hydrogen bond between them. A similar lid allowed the substrate to be better placed in the active pocket, thereby increasing the catalytic efficiency of the enzyme. The mutated amino acid Asp259 still formed hydrogen bonds with the surrounding amino acids Asp257 and Arg 261, as in the wild-type (Fig. 8b-c).

### Mass spectrometry and activity determination of LMWH

To study the degradation of heparin by the enzyme after mutation, we performed mass-spectrometric detection and activity measurement on the degradation products. As shown in Additional file 3: Figure S3, we measured the mass spectra of the enzymatic hydrolysates of wild-type, S169D, A259D and S169D/A259D. Compared with the standards of LMWH, the enzymatically hydrolyzed products we obtained met the standard requirements. The molecular masses of the products were 4000–6000 Da. The anti-FXa and anti-FIIa activities of the enzymatic hydrolysate were determined by the chromogenic substrate method, and the results are shown in Table 3. The anti-FXa and anti-FIIa activity of the enzymatic hydrolysate met the requirements of the pharmacopoeia standard at 1.5–2.5 [28].

**Table 3:** Anticoagulant activity and molecular weight of LMWH

Sample	Anti-FXa(IU/mg)	Anti-FIIa(IU/mg)	Anti-FXa/Anti-IIa	Relative molecular mass
1	121.72 ± 4.18	48.37 ± 5.02	2.5164 ± 0.15	4326.984
2	112.18 ± 9.32	59.01 ± 3.21	1.9010 ± 0.06	5168.031
3	113.24 ± 7.51	56.35 ± 6.08	2.0096 ± 0.13	4944.875
4	115.72 ± 3.29	58.92 ± 3.72	1.9640 ± 0.09	5209.882
5	119.89 ± 2.87	57.05 ± 3.02	2.1015 ± 0.12	5032.487

1:Low molecular weight heparin, 2:Heparin degraded by wild-type Ph-hepI,3:Heparin degraded by mutant S169D,4:Heparin degraded by mutant A259D, 5:Heparin degraded by mutant S169D/A259D

## Discussion

Heparinase I is used for the production of low-molecular-weight heparin (LMWH) from heparin, and the LMWH can retain the anticoagulant properties [32]. There are many sources of heparin, and the porcine intestinal mucosa is the most important source [33], but the low production of LMWHs limits its development. The major degradation methods of heparin include chemical lysis and enzymatic lysis [34]. Although chemical lysis is simpler than enzymatic lysis, it has many disadvantages. The use of nitrite in the lysis process has caused serious environmental pollution, and the drug activity of prepared LMWHs is low. However, LMWH production still requires the use of chemical degradation instead of purely enzymatic degradation due to the low production and activity of enzymes [35]. Natural HepI is mainly produced by *Pedobacter heparinus* [36]. Since the yield of HepI in *P. heparinus* is low, recombinant expression of HepI using genetic engineering strategies has received widespread attention [16]. However, recombinantly expressed HepI in *E. coli* easily aggregates into insoluble inclusion bodies [10]. To solve this problem, fusion expression with a tag that has the ability to improve the formation of the correct conformation is a useful approach for the soluble expression of this protein [37–39]. In our previous research, to establish a soluble expression system of Ph-HepI, the *hepI* gene (coding for HepI) was fused with a hexahistidine-tag (6 × His) and genes encoding a small ubiquitin-like modifier (SUMO), a flexible peptide linker (G4S) and a bovine enterokinase site (D4K) [24].

By fusion to maltose binding protein (MBP), the first soluble expression of HepI was achieved in *E. coli* [39]. The soluble expression of HepI has been greatly improved by fusioing to a SUMO-tag, but the enzyme activity is still very low [40]. Therefore, this study improved the specific enzyme activity by using homology modeling, multiple sequence alignment, molecular docking, and site-directed mutagenesis.

The molecular modification of heparinase is important to improve the performance of the enzyme. Substitution of Cys297 to serine in MBP-HepI offered a 30.6% increase in recovered total enzyme activity due to a mutation-induced thermostabilizing effect [31]. The clone *E. coli*-heparinase-I133/P316 with two amino acid substitutions has been screened and identified by error-prone PCR. Compared with the wild-type, the mutant HepI has a 57.8% increased enzyme activity [41]. In these studies, the HepI enzyme activity was improved by directed evolution and fusion expression, but these mutations were not well explained in the HepI structure.

To date, there is no crystal structure of Ph-HepI for reference. Therefore, our focus was to explore the structure and function of HepI through multisequence alignment and 3D homologous modeling (Fig. 2). Since Ph-HepI shared 62.5% identity with structurally known Bt-HepI from *Bacteroides thetaiotaomicron* (Fig. 1), a series of active residues of Ph-HepI were predicted (E87, Y89, D169, K199, E248, W254, D259, N351, and D352) through multiple-sequence alignment, homology modeling and molecular docking. Based on sequence alignment of Ph-HepI and Bt-HepI, the residues S169 and A259 in the Ph-HepI were found to be different from in the Bt-HepI, in which they were instead of D155 and D253 (Fig. 3). Three mutants (S169D, A259D, and S169D/A259D) were constructed by site-directed mutagenesis and analyzed for their enzymatic properties. Compared with the wild-type, the specific enzyme activity of the three mutants (S169D, A259D, and S169D/A259D) was increased by 50.18, 40.43, and 122.05%, respectively (Fig. 6). The optimum reaction temperature and pH of the three mutants were 37 °C and 7, which was consistent with the wild-type. However, the optimal concentration of Ca^2+^ for wild-type and A259D was 10 mM, while the optimal concentration of Ca^2+^ for S169D and S169D/A259D was 50 mM (Fig. 7).

Our study found that the exchange of several amino acids could produce more active enzymes, which had basically identical interresidue interactions to that observed in the Bt-HepI crystal structure. The exchange of S169 with aspartic acid resulted in an enzyme with higher activity, which promoted D169 to become the nucleophilic amino acid of Ph-HepI and resulted in its combination with calcium. This is consistent with the structure of heparinase I binding to calcium, which can increase the initial activity of Ph-HepI [31, 42]. When Ser was mutated to Asp in Ph-HepI, the enzyme activity was significantly improved. The underlying cause of increased enzyme activity may be the binding and activation of D169 with the calcium ion and the formation of new hydrogen bonds with nearby amino acid residues.

However, the amino acid at position 259 was Ala instead of Asp. Alanine is hydrophobic and did not form a hydrogen bond with Tyr89, but aspartic acid has a side-chain radical containing a carboxyl, which allowed it to produce hydrogen bond with Tyr89. Following the construction of the combined mutation (S169D/A259D), the specific enzyme activity had obvious improvements. The mutants only specifically increased the specific enzyme activity of the enzyme and did not change the enzyme degradation substrate. Altogether, our results provide structural information to reveal Ph-HepI’s catalytic mechanisms and guide rational engineering of Ph-HepI for industrial applications.

## Conclusions

In summary, the enzymatic activity of Ph-HepI was significantly improved through site-directed mutagenesis with the help of a rational structure-based design method. Compared with the wild-type enzyme, the enzyme activity and catalytic efficiency (*k*cat/*K*m) of the mutant S169D/A259D increased by 122.05 and 406.18%, respectively. We provided an effective method for the modification of heparinase I and laid the foundation for the industrial application of heparinase I.

## Additional files

Additional file 1: **Figure S1.** Phylogenetic tree of characterized heparinase I of PL13 family (PDF 113 kb)

Additional file 2: **Figure S2.** The stereochemical spatial arrangement of amino acid residues in the modelled 3D structure of Ph-HepI in the favored region of the Ramachandran plot (PDF 106 kb)

Additional file 3: **Figure S3.** Mass spectrometry analysis of degradation products (PDF 92 kb)

## Data Availability

The datasets used and/or analyzed during the current study are available from the corresponding author on reasonable request.

## Abbreviations

Bt-HepI: Heparinase I from *Bacteroides thetaiotaomicron*
HepI: Heparinase I
Ala: Alanine
Asp: Aspartic
*E. coli*: *Escherichia coli*
HepI: Heparinase I
IPTG: Isopropyl β-D-thiogalactoside
LB: Luria-Bertani
LMWH: Low molecular heparin
Ph-HepI: Heparinase I from *Pedobacter heparinus*
PL13: Polysaccharide lyases family 13
Ser: Serine
