# Supplementary figures and images for: Structure-based engineering of heparinase I with improved specific activity for degrading heparin
Source: BMC Biotechnol. 2019 Aug 9;19:59. doi: 10.1186/s12896-019-0553-3 (PMC6688311; doi:10.1186/s12896-019-0553-3)

**Figure S1** Phylogenetic tree of characterized heparinase I of PL13 family.

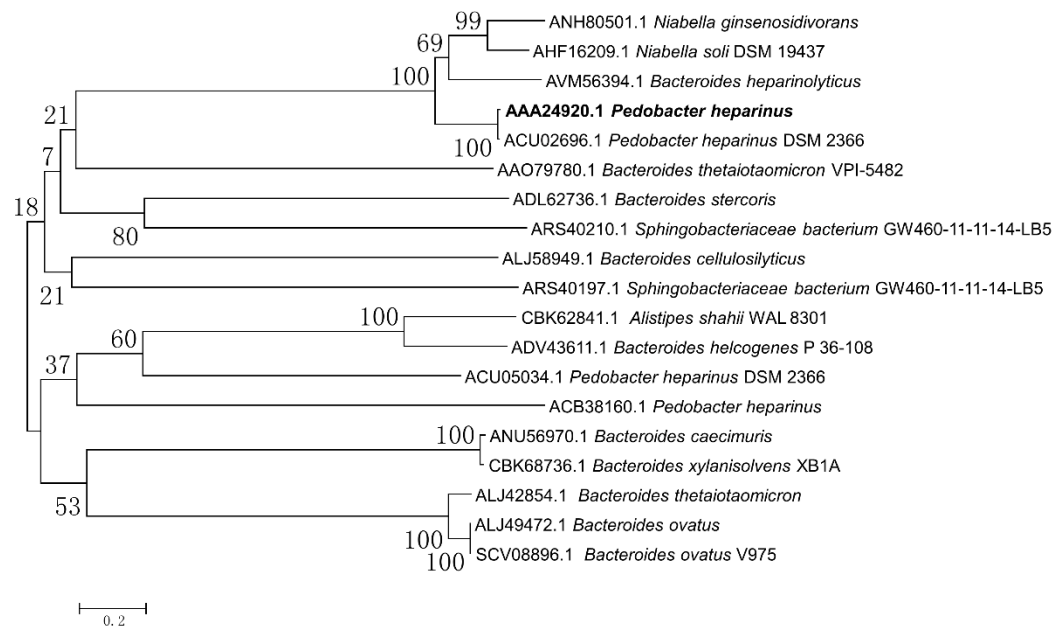

Supplement: Supplementary file 1 — Figure S1. Phylogenetic tree of characterized heparinase I of PL13 family (PDF 113 kb) [file 12896_2019_553_MOESM1_ESM.pdf]
