# Supplementary material for: Structure-based engineering of heparinase I with improved specific activity for degrading heparin
Source: BMC Biotechnol. 2019 Aug 9;19:59. doi: 10.1186/s12896-019-0553-3 (PMC6688311; doi:10.1186/s12896-019-0553-3)

Figure S2 The stereochemical spatial arrangement of amino acid residues in the modeled 3D structure of Ph-HepI in favored region of the Ramachandran plot.

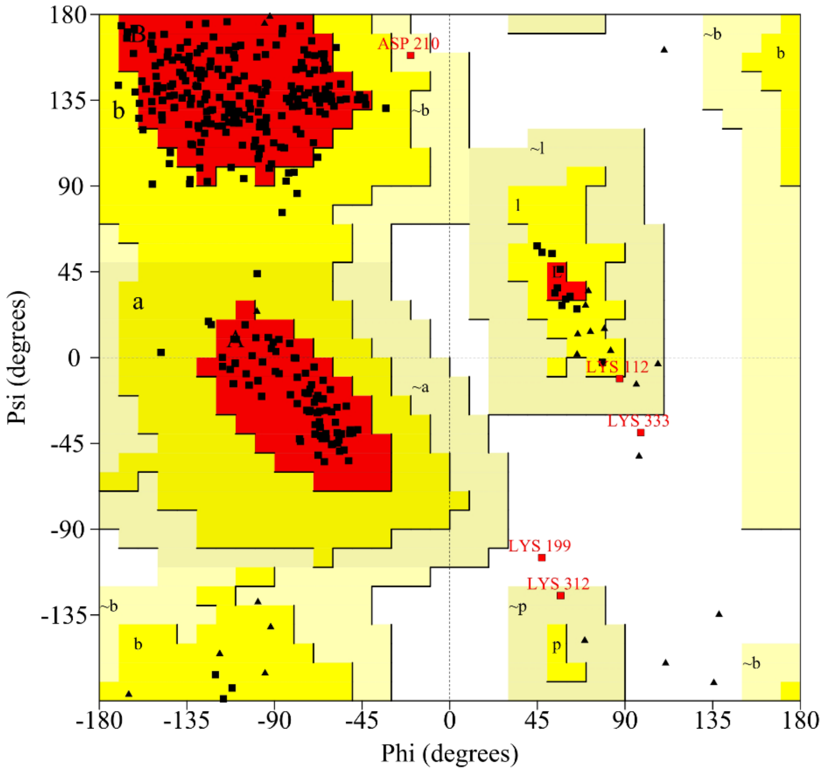

Supplement: Supplementary file 2 — Figure S2. The stereochemical spatial arrangement of amino acid residues in the modelled 3D structure of Ph-HepI in the favored region of the Ramachandran plot (PDF 106 kb) [file 12896_2019_553_MOESM2_ESM.pdf]
