# Supplementary material for: Structure-based engineering of heparinase I with improved specific activity for degrading heparin
Source: BMC Biotechnol. 2019 Aug 9;19:59. doi: 10.1186/s12896-019-0553-3 (PMC6688311; doi:10.1186/s12896-019-0553-3)

Figure S3 Mass spectrometry analysis of degradation products. (a) LMWH standard; (b) Degradation product of Wild-type enzyme; (c) Degradation product of mutant enzyme (S169D); (d) Degradation product of mutant enzyme (A259D); (e) Degradation product of mutant enzyme (S169D/A259D).

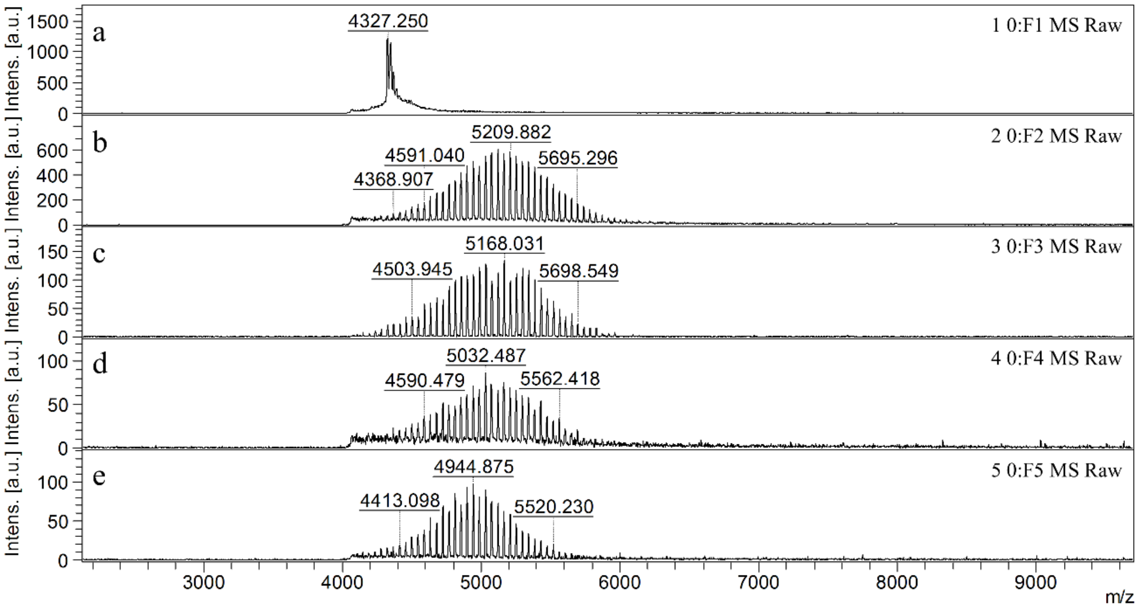

Supplement: Supplementary file 3 — Figure S3. Mass spectrometry analysis of degradation products (PDF 92 kb) [file 12896_2019_553_MOESM3_ESM.pdf]
